# A public mid‐density genotyping platform for pecan [*Carya illinoinensis* (Wangenh.) K. Koch]

**DOI:** 10.1002/tpg2.70262

**Published:** 2026-06-13

**Authors:** Shufen Chen, Meng Lin, Dongyan Zhao, Warren Chatwin, Xinwang Wang, Angelyn Hilton, John T. Lovell, Jennifer Randall, Kasia Heller‐Uszynska, Cristiane H. Taniguti, Craig T. Beil, Moira J. Sheehan

**Affiliations:** ^1^ Breeding Insight Cornell University Ithaca New York USA; ^2^ Breeding Insight Institute of Food and Agricultural Sciences, University of Florida Gainesville Florida USA; ^3^ USDA‐ARS Crop Germplasm Research Unit College Station Texas USA; ^4^ Genome Sequencing Center HudsonAlpha Institute for Biotechnology Huntsville Alabama USA; ^5^ Department of Entomology, Plant Pathology and Weed Science New Mexico State University Las Cruces New Mexico USA; ^6^ Diversity Arrays Technology Canberra Australian Capital Territory Australia

## Abstract

Pecan [*Carya illinoinensis* (Wangenh.) K. Koch] is the fifth‐largest tree nut in global cultivation, with 80% of production occurring in the southern states of the United States. Despite the economic and health benefits of pecans, there is a lack of genomic tools available to breeders for crop improvement. The pecan breeding community is small, and most breeding programs have many barriers to adopting technology, particularly in the cost and know‐how needed to create and use genetic marker panels for genomic‐based decisions in selection. Here, we report the creation of a DArTag (Diversity Array Technology [DArT]) panel of 3100 loci distributed across the diploid pecan genome for use in molecular breeding and genomic prediction. Here, we show the panel's ability to distinguish key parents and founders of cultivated pecan from other *Carya* species that may be used in pre‐breeding. We also demonstrate that the resultant data can create a linkage map in a biparental population. The creation of this marker panel brings cost‐effective and rapid genotyping capabilities to pecan breeding programs, making routine genotyping a reality for any pecan breeder. Furthermore, the open access provided by this platform enables the comparison and integration of genetic datasets generated on the marker panel across projects, institutions, and countries.

## Core Ideas

- The United States is the largest producer of pecans, producing about 80% of the global supply.
- Pecan has a long juvenile period of 7–10 years before nuts are produced.
- There is a lack of genomic tools for pecan breeders to use in crop improvement.
- The 3K DArTag panel provides affordable access to genomic data for breeders.
- Genomic data could accelerate predictive breeding and selection in pecan.

## Plain Language Summary

Pecan is an important nut tree native to the United States and Mexico. The United States produces 80% of the global supply of pecans, and the USDA‐ARS has several research programs to preserve genetic diversity and improve pecan varieties for farmers. Crop improvement of pecan has lagged behind other crops, in part due to its long juvenile period (7–10 years from nut to nut) and the lack of genomic tools. We created a genetic marker panel of 3100 loci across the genome that is easy and affordable for pecan breeders to obtain genetic data from their breeding materials. The resulting data can be used to build linkage maps, perform quantitative trait loci studies, conduct genome‐wide association studies, and perform genomic prediction. Predictive breeding techniques could allow breeders to make breeding and selection decisions much sooner based solely on genetic information.

**Abbreviations**
AFLP: amplified fragment length polymorphism
Alt: alternative allele
AltMatch: target variant site matches alternative
*BLAST*
: basic local alignment sequence tool
*EST*
: expressed sequence tag
*FAST*
: Fast‐All
GBS: genotyping‐by‐sequencing
GWAS: genome‐wide association study
indel: insertion and deletion site
LG: linkage group
MADC: missing allele discovery count
MAS: marker‐assisted selection
PC: principal component
PCA: principal component analysis
PIC: polymorphism information content
QTL: quantitative trait locus
RAPD: random amplified polymorphic DNA
Ref: reference allele
RefMatch: target variant site matches reference
SNP: single‐nucleotide polymorphism
SSR: simple sequence repeat

## 1 INTRODUCTION

Molecular techniques have been employed for nearly four decades to enhance and speed the breeding efforts for major staple food crops like tomato (*Solanum lycopersicum* L.), maize (*Zea mays* L.), and barley (*Hordeum vulagare* L.) (Feuerstein et al., 1990; Hasan et al., 2021; Helentjaris et al., 1985; Tanksley, 1983). Over time, molecular biology techniques have been paired with high‐quality phenotypic data to perform quantitative trait locus (QTL) mapping, genome‐wide association study (GWAS), genomic selection, and prediction, further fueling breeding for quantitative or complex traits (Eathington et al., 2007; Heffner et al., 2009; Helentjaris et al., 1985; Lorenzana & Bernardo, 2009). While these achievements are significant, many crop species grown for human consumption remain unable to apply these techniques in their breeding efforts. Many breeders would like to adopt molecular breeding tools and techniques, but sometimes, doing so is hampered by considerable barriers to entry. The range of barriers, and how surmountable they are, varies from species to species and is impacted by species‐specific challenges in logistics, technical know‐how, biology, and the growing environment.

Pecan [*Carya illinoinensis* (Wangenh.) K. Koch] is an economically important and agriculturally significant native nut tree in North America (Grauke et al., 2003; Helentjaris et al., 1985). Its use as a food crop predates the colonization of the United States, and the name “pecan” comes from the Algonquin language (likely Illinois or Ojibwe) term pakani or paka:ni describing nuts that require a stone to crack (Mitchell, 2020). In 2023, the United States produced 80% of the world's pecan yield on an estimated 441,000 acres (National Agricultural Statistics Service [NASS], 2024). Pecan has a diploid genome with 16 chromosomes, a monoecious reproduction system, and is a long‐lived and perennial plant cultivated for its culinary, ornamental, and lumber uses (Bentley et al., 2020). In 2023, US pecan production reached approximately 271 million pounds, with a total market value of 460 million. Improved variety pecan production reached 258 million pounds (117 million kg), which accounted for 95% of the United States' total pecan production (National Agricultural Statistics Service [NASS], 2024). The development of improved cultivars via phenotypic evaluation and selection is challenging due to inbreeding depression, long juvenile periods, a complicated flowering pattern, long nut maturation times, and mechanical management and harvest systems that can damage trees over time (Bentley et al., 2020). Several molecular marker systems have been developed for pecan breeding, including tens of simple sequence repeat (SSR) (Grauke et al., 2003; Zhang et al., 2020), hundreds of amplified fragment length polymorphism (AFLP) (Beedanagari et al., 2005; Bentley et al., 2020; Vendrame et al., 1999), hundreds of random amplified polymorphic DNA (RAPD) (Beedanagari et al., 2005; Conner & Wood, 2001), and thousands of single nucleotide polymorphisms (SNPs) (Bentley et al., 2020). Most of the platforms above have been utilized to some extent in the development of marker‐based breeding strategies for pecan. The first molecular‐marker‐based linkage map was created with RAPD and AFLP (Beedanagari et al., 2005), a GWAS based on genotyping‐by‐sequencing (GBS) (Bentley et al., 2019, 2020), and a linkage map and QTL analysis also based on GBS (Bentley et al., 2020). Additionally, the availability of genome assemblies and gene annotations for pecan, as well as pan‐genome integration, has been reported to accelerate pecan tree breeding (Lovell et al., 2021).

Next‐generation sequencing technologies have been used to detect large numbers of SNP markers (millions to tens of millions) genome‐wide from germplasm collections and structured populations, which have then been employed in QTL analysis, GWAS, and genomic selection (Plomion et al., 2016). Most SNP data in pecan studies to date were derived from a GBS technique (Elshire et al., 2011). Since GBS involves short‐read sequencing of a reduced genome representation, merging datasets is often difficult or even impossible due to the high level of missing data in GBS. These missing data primarily result from the lack of reproducibility in sequencing the same loci across different runs in all samples, making GBS less effective for recurrent selection and marker‐assisted selection (MAS) breeding applications.

For breeding applications, breeders require the ability to detect the same loci in all samples and over several generations of selection to monitor introgressions and linkage drag, as well as determine parentage. Counter to expectations, the millions of SNPs generated by GBS prove unwieldy for many breeders to use in selection decisions. Many breeders prefer a more tractable system that has fewer markers, at consistent sites, with low missing data. Unlike GBS, proprietary targeted amplicon‐based genotyping technologies such as DArTag (Diversity Array Technology [DArT]), Flex‐Seq (RAPiD Genomics), and Capture‐Seq (LGC Genomics), among others, have low missing data rates and query the same exact loci in all samples across all genotyping projects, allowing new data to be easily appended to existing data (Darrier et al., 2019; Telfer et al., 2019; Wang et al., 2020). The amount of data returned is in the tens of thousands, rather than the millions of reads from GBS, simplifying downstream bioinformatics processing (Darrier et al., 2019; Milner et al., 2018; Telfer et al., 2019; Wang et al., 2020). This, in turn, speeds up the analysis time for MAS, introgression tracking, linkage mapping, GWAS, and genomic prediction (Darrier et al., 2019).

Here, we developed and tested a DArTag panel comprising 3100 loci distributed across the pecan genome to provide a low‐cost, mid‐density marker genotyping solution suitable for pecan breeding. DArTag is an oligo‐hybridization to an amplicon‐based targeted genotyping platform developed by DArT (Kilian et al., 2012). Oligos are custom‐designed to target known genetic variants (SNPs and indels [insertion and deletion site] <20 bp) and their flanking genomic regions, yielding sequencing products of 81–109 bp. The 3K DArTag panel offers a novel genetic resource to the pecan breeding community, balancing cost, data complexity, repeatability, and versatility for several common downstream analyses in molecular breeding and genomic prediction applications.

**Core Ideas**

- The United States is the largest producer of pecans, producing about 80% of the global supply.
- Pecan has a long juvenile period of 7–10 years before nuts are produced.
- There is a lack of genomic tools for pecan breeders to use in crop improvement.
- The 3K DArTag panel provides affordable access to genomic data for breeders.
- Genomic data could accelerate predictive breeding and selection in pecan.

## 2 MATERIALS AND METHODS

### 2.1 SNP discovery and selection of 3K marker loci for a DArTag panel

An SNP database of 181,589,925 SNPs generated from a diversity panel of 48 *C. illinoinensis* pecan accessions was most frequently used for making crosses (Table S1). The SNP database was created by aligning reads derived from the study by Grabowski et al. (2025) to the reference genome *C. illinoinensis* v1.1 (Lovell et al., 2021). A high confidence set of 13,918 SNPs (Figure S1A) were obtained mainly using vcftools v0.1.16 (Danecek et al., 2011) by requiring (1) a quality score >20, (2) biallelic state with at least two reads for the reference allele and two for the alternative allele, (3) a minor allele frequency >5%, (4) a missing rate <25%, (5) the linkage disequilibrium pruned at *r* ^2^ < 0.9, (6) location in low polymorphic region (i.e., location at least 30 bp downstream or upstream from another marker), (7) prioritization of SNPs in non‐repetitive regions within genic regions, and (8) a nonsignificant Hardy–Weinberg equilibrium test value at *p*‐value of 0.01. The set of 13,918 markers was submitted to DArT for quality control using its proprietary protocols, and 12,817 passed DArT's testing. Breeding Insight made a further selection to target even genomic distribution and to minimize gaps across the genome to identify the final 3100 SNPs, which included 30 markers potentially associated with agronomic traits (Chatwin, personal communication, 2024) (Figure S1B). Oligos were designed at DArT and synthesized by Integrated DNA Technologies, Inc.

### 2.2 Test samples and genotyping results

A set of 376 pecan lines representing 16 species (details shown in Table S1) was selected for genotyping and validation of the 3K DArTag panel (Pecan_DArTag_BI_Cornell_University [1.0]). The set included a diverse collection of 186 accessions and a biparental population of 190 F_1_s including the two parents (Table S1). Their leaf tissues were collected and sent to DArT, where DNA extraction, library preparation, and sequencing were conducted using its proprietary protocols. The resulting genetic data were provided by DArT in a missing allele discovery count (MADC) file. The MADC included all the read counts for all detected microhaplotypes (81 bp short sequences) identified through the consensus of 81 bp amplicons across the 3K marker loci. These microhaplotypes encompassed the target SNPs as per assay design and off‐target SNPs (i.e., discovery variants), producing multi‐allelic genetic data for each locus. For clarity, microhaplotypes matching the reference and variant at the target SNP sites were denoted as reference (Ref) and alternative (Alt) microhaplotypes, respectively. Those containing off‐target SNPs were denoted as target variant site matches reference (RefMatch) or target variant site matches alternative (AltMatch). To ensure consistency within and between projects, unique and fixed microhaplotype names (i.e., AlleleIDs) were established. This was particularly important due to duplicate RefMatch and AltMatch names in the raw MADC files provided by DArT. To retain and assign fixed AlleleIDs, all the RefMatch and AltMatch microhaplotypes were filtered in R software v4.5.2 (R Core Team, 2025), requiring reads in at least 10 samples, each with a minimum of two reads. The retained microhaplotypes were then queried against the most recent microhaplotype database (a fasta file containing all the microhaplotypes identified in any and all previous projects) using the National Center for Biotechnology Information basic local alignment search tool (BLAST, v2.11.0+). Only those microhaplotypes with ≥90% identity and over ≥90% coverage were kept and assigned fixed AlleleIDs. For DArTag loci with ≥10 microhaplotypes (potentially indicating paralogous sequence amplification), only Ref and Alt alleles were retained. The final raw dataset comprised 10,559 microhaplotype alleles from 3100 DArTag loci across 376 samples.

### 2.3 Marker detection rate

To evaluate the detection rate of the 3K DArTag loci, total read depth was calculated for each of the 376 lines and for each of the 3100 loci. A marker locus with <10 reads in any of the 376 lines was considered as missing data. The sample‐based missing rate was calculated as the number of missing loci over the number of tested loci (*n* = 3100). Only one sample from the diverse population was removed due to its high missing rate (≥95%). After removing the single sample with a high missing rate, 3073 marker loci, including 10,199 microhaplotypes that were successfully amplified (≥10 reads in ≥10 samples each), were retained, reflecting a missing data rate of 0.9%.

### 2.4 Principal component analysis

Principal component analysis (PCA) was performed using read count data of 3073 loci for the 375 tested samples. Allele frequency at target SNP loci was approximated using the ratio of the reference (Ref + RefMatch) read depth to the total (Ref + Alt + RefMatch + AltMatch) read depth. PCA was performed using the estimated allele frequency matrix using the AddPCA function in polyRAD v2.0.0 (Clark et al., 2019) and visualized using the R‐package ggplot2 v3.5.1 (Wickham, 2016).

### 2.5 Variant sites extraction

As mentioned above, the MADC microhaplotypes frequently include additional variants beyond the initial assay design sites and provide more genetic discrimination of multiple alleles. To identify all variants, RefMatch and AltMatch sequences were aligned with their respective reference and alternative microhaplotypes using the Bio.pairwise2 from BioPython v1.7.9 (Cock et al., 2009). Variant sites were extracted from the pairwise alignments. A total of 7547 SNPs, including both target and off‐target SNPs, were extracted from 10,199 microhaplotypes of the 3073 amplified marker loci across the entire set of validation germplasm. Read depths of each variant site were calculated by collapsing read depths of all sequences that contained that genomic variant.

### 2.6 Polymorphism information content in the diverse population

Polymorphism information content (PIC) was estimated for the diversity population using both microhaplotype and SNP read counts. A total of 10,199 microhaplotypes from 3073 DArTag loci were used for microhaplotype‐based PIC evaluation. The frequency of each microhaplotype was approximated using the ratio of the microhaplotype‐associated read counts to the total read counts of its corresponding locus across the 185 diversity samples. SNP‐based PIC was estimated using the same formula as microhaplotype, and 7547 SNPs from 3073 DArTag loci were used for SNP‐based PIC evaluation. Allele frequency was estimated using read depth associated with each allele in an SNP. PICs were calculated for each DArTag locus and SNP for the diverse population using the following formula described by (Botstein et al., 1980):

$$\mathrm{PIC}=1-\sum_{i=1}^{n}p_{i}^{2}-\sum_{i=1}^{n-1}\sum_{j=i+1}^{n}2\;p_{i}^{2}p_{j}^{2},$$

where *n* is the number of alleles, $p_{i}$ is the frequency of the *i*th allele, and $p_{j}$ is the frequency of the *j*th allele.

### 2.7 Dosage calling of samples using genotyping results

Genotype dosage calling was performed for 7547 SNPs, which included both target and off‐target SNPs for the F_1_ and diversity populations separately using the “multidog” function with the “f1” and “norm” models, respectively, with the ploidy level set to 2 in the R‐package updog v2.1.5 (Gerard et al., 2018). The parental genotypes were specified for the F_1_ population. After dosage calling, SNPs were filtered for the estimated allele bias (0.05 < bias < 2), the estimated proportion of individuals misclassified (prop_mis < 0.05), and the estimated overdispersion parameter (od < 0.05). A total of 6520 SNPs were retained for the diversity population and 7344 SNPs for the F_1_ population. An SNP was considered informative if it had at least two different genotypes present among the evaluated accessions.

### 2.8 Linkage map construction

A total of 7344 SNPs were used to initiate the genetic map construction for the F_1_ population using MAPpoly2 v2.0.0 (Mollinari & Garcia, 2019). Nonconforming markers (*n* = 5761) that were monomorphic (*n* = 5132) and other unexpected segregation (e.g., aa × bb, bb × aa segregation) (*n* = 629) markers, and redundant markers that had identical mapping information to a mapped marker (*n* = 414) were removed from the subsequent analysis. No markers or individuals were removed based on a missing rate of >2%. However, markers (*n* = 55) deviating from the Mendelian segregation ratio at Bonferroni adjusted *p*‐value of 0.05 were removed. Suspicious F_1_ individuals (*n* = 6) that did not appear to be derived from the specified parent cross were also excluded prior to mapping (Figure S2). Pairwise recombination frequencies were computed for the remaining 1114 markers and 182 F_1_ individuals using the “*pairwise_rf*” function. A total of 16 linkage groups (LGs) (consistent with expectations for 16 chromosomes) were constructed based on the recombination fraction matrix. For each LG, the “*pairwise_phasing*” function was used, together with markers’ genomic orders (physical position), to perform phasing. Map was constructed using both parents simultaneously by setting parent = “p1p2” in the “*mapping*” function.

## 3 RESULTS

### 3.1 Performance on the 3K pecan DArTag genotyping panel

**FIGURE 1:**
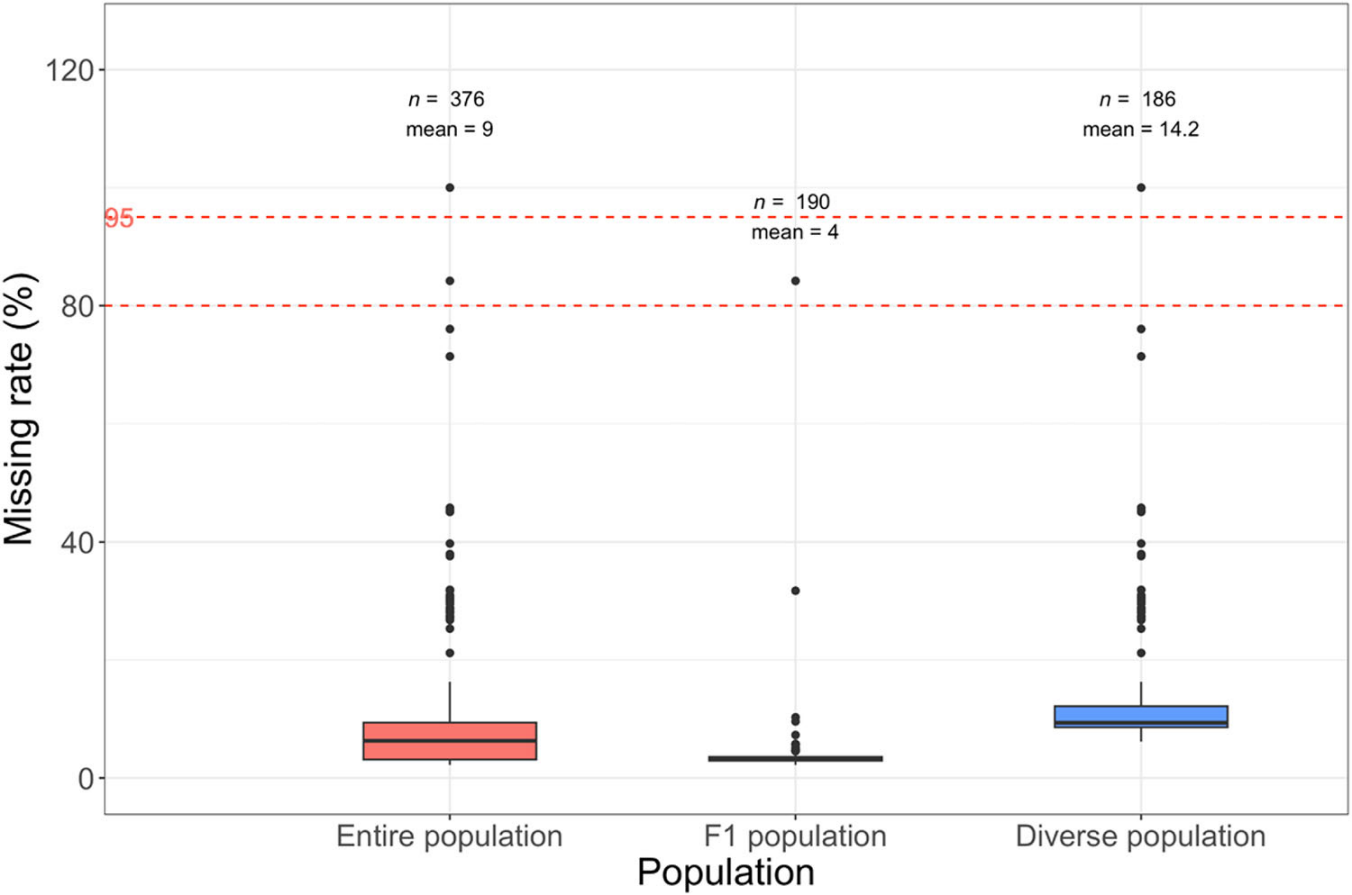
Sample‐based missing data rates of the testing materials for the 3K pecan DArTag panel. For each population evaluated, “*n*” is the number of samples, and “mean” is the average missing data rate. Two dotted lines (red) indicate the 80% and 95% missing data rates.

The performance of the 3K pecan DArTag marker panel was evaluated on a set of 376 diploid pecan accessions, including an F_1_ population and a genetically diverse population. The DArTag MADC report contains all microhaplotypes (81 bp sequences) detected from the validation sample set. Of the 10,559 microhaplotypes detected, 6200 are Ref and Alt microhaplotypes, and 4359 are microhaplotypes containing off‐target sequence variants. Overall, the 3K panel showed high detection efficiency. The average sample‐based missing rate of the entire population was 9.0% (Figure 1), with 90% (*n* = 338) of total samples having amplicons from ≥80% marker loci (Table S2). In each of the individual populations, the F_1_ population had a lower average missing rate of 4.0% (2.2%–84.2%) than the average missing rate of 14.2% (6.2%–100%) in the diversity panel (Figure 1; Table S3).

### 3.2 Principal component analysis reveals distinct genetic patterns in pecan populations

**FIGURE 2:**
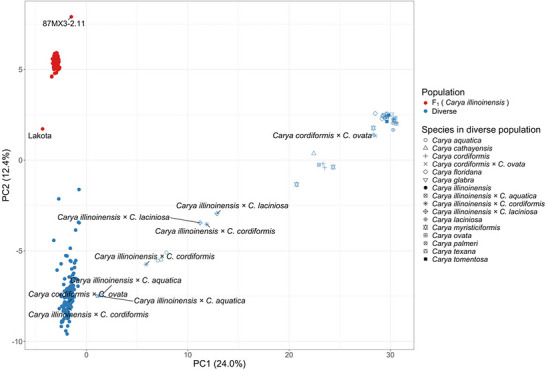
Principal component analysis (PCA) using read counts of all 375 validation accessions. Lakota and 87MX3‐2.11 are parents of the F_1_ population. PC, principal component.

To assess the genetic diversity and population structure among 375 pecan samples, PCA (Table S1) was conducted using the read counts. The analysis revealed that the first two principal components (PCs) collectively accounted for 36.4% (PC1 24.0% and PC2 12.4%) of the total genetic variance in the tested materials (Figure 2). The PCA plot (Figure 2) revealed distinct patterns of genetic relationships among the samples. The F_1_ population exhibited a tight clustering pattern, positioned intermediately between their parental cultivars Lakota and 87MX3‐2.11. This clustering pattern is typical of a biparental F_1_ population and confirms the expected genetic inheritance pattern, where offspring inherit approximately equal genetic contributions from both parents.

In contrast, the diversity samples composed of key *C. illinoinensis* founders and parents as well as related *Carya* species and interspecific hybrids demonstrated considerable variation in their distribution across the plot. Most of the accessions (*C. illinoinensis*) exhibited a relatively tight cluster. For the related *Carya* species, there were fewer accessions included in the study (Table S1); however, those accessions belonging to the same species clustered close to one another, while accessions from different species were relatively far apart, showing a wide dispersion on the plot. Additionally, hybrids resulting from mating between different *Carya* species are generally positioned intermediately between their parental species, except for two hybrids that clustered with *C. illinoinensis* accessions, which may indicate they are mislabeled and not true interspecific hybrids. These patterns reflect the broad genetic base of the diversity collection and validate the marker panel's ability to detect and represent known genetic relationships for hybrids within and between species, despite being designed for breeding for only *C. illinoinensis*.

### 3.3 Polymorphism information content for the DArTag loci and SNPs in the diverse population

**FIGURE 3:**
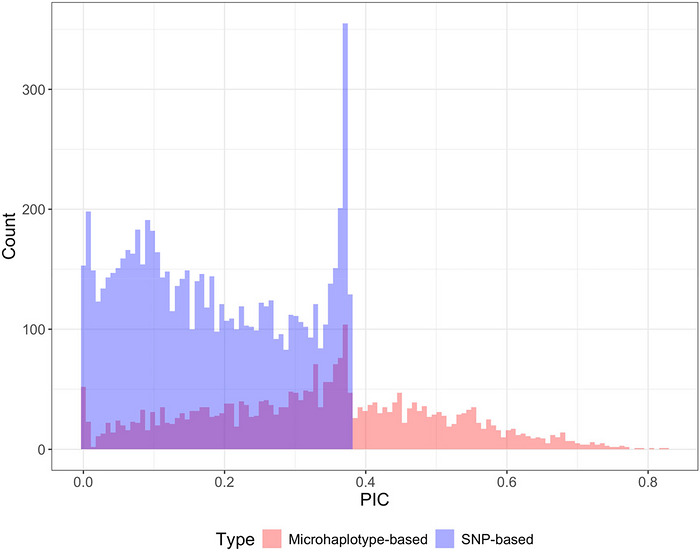
Polymorphism information content (PIC) values were estimated using 10,084 microhaplotypes and 7443 single nucleotide polymorphisms (SNPs) in the diverse population.

The DArTag loci and SNPs with a PIC value of zero based on read counts were removed as monomorphic markers in the diverse population. Microhaplotype‐based PIC was estimated for 3016 DArTag loci including 10,084 microhaplotypes. The number of microhaplotype per loci varied from two to nine with the average microhaplotype frequencies ranging from 0.1 to 0.5, as loci with >10 microhaplotypes were excluded (see Section 2). The microhaplotype‐based PIC values ranged from 0.0001 to 0.825, with an average of 0.334 (Figure 3; Table S4). The SNP‐based PIC values were estimated for 7442 SNPs and ranged from 0.0001 to 0.375, with an average of 0.182 (Figure 3; Table S5). Additionally, microhaplotype‐based PIC from 2968 DArTag loci, including 9897 microhaplotypes, were compared with SNP‐based PIC using polymorphic target SNPs extracted from those loci. Over 67% of the loci showed increased PIC using microhaplotype read counts (Figure S3). These results indicate that microhaplotypes generally contain more genetic variability compared to single SNPs, highlighting their potential utility in capturing genetic diversity in breeding and genomic studies.

### 3.4 Distribution and comparison of sequence polymorphisms in the F_1_ and diverse pecan populations

Analysis of microhaplotype distribution revealed distinct patterns between the F_1_ and diverse populations. After removing samples with high missing data rates and non‐amplified loci, 7229 of 10,199 microhaplotypes were detected in the F_1_ population and 9849 in the diverse collection. Similar patterns were observed at the SNP level. The 7547 SNPs were extracted from 10,199 microhaplotypes. After dosage calling, 6232 out of 6520 SNPs were informative in the diversity collection, while 1573 out of 7344 SNPs were informative in the F_1_ population. The higher number of microhaplotypes and SNPs observed in the diverse collection aligns with expectations, as the diversity population encompasses a broader genetic base.

### 3.5 Creation of a linkage map in pecan

**FIGURE 4:**
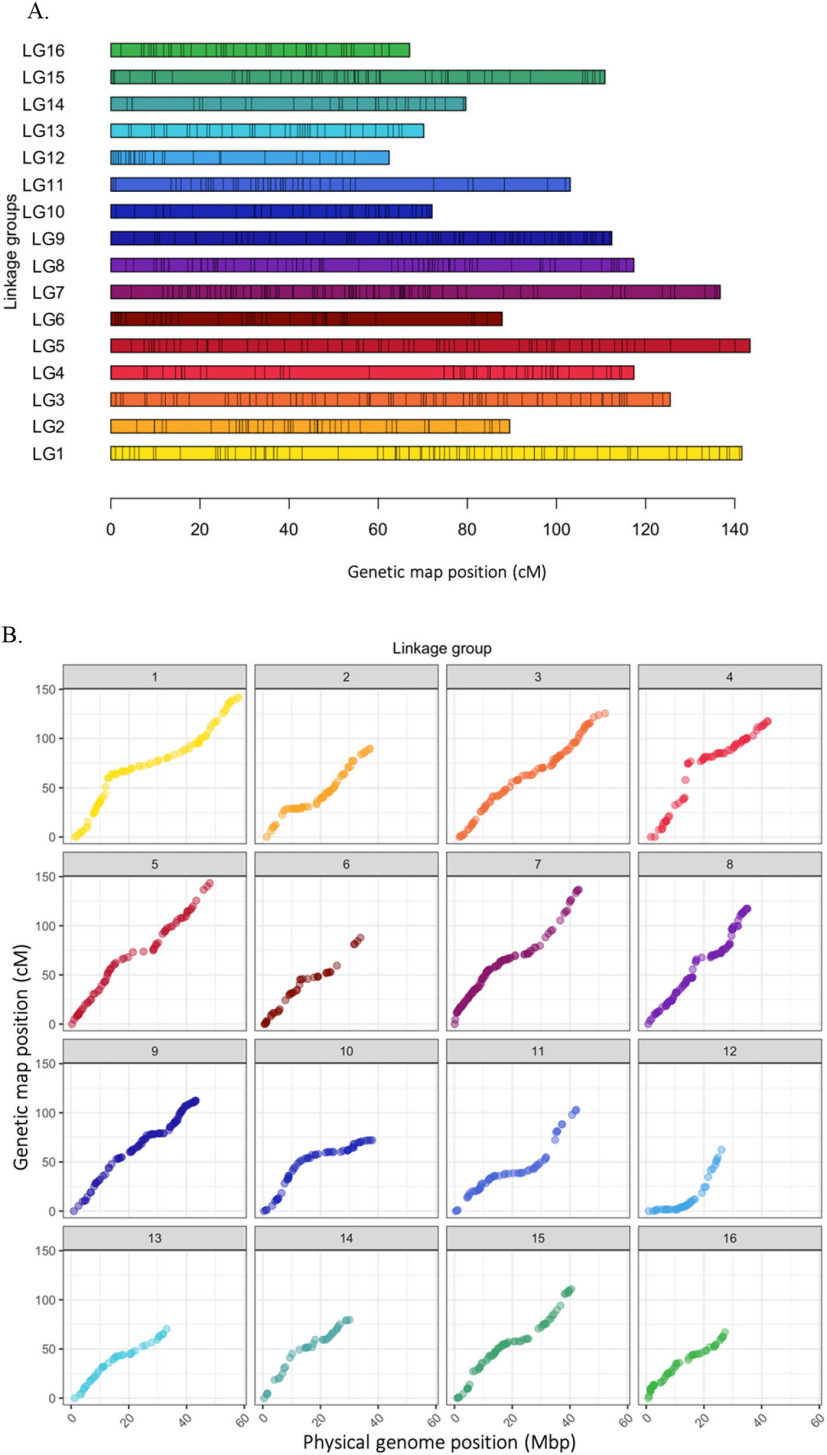
De novo linkage map for an F_1_ population derived from Lakota and 87MX3‐211 (A) Genetic map of the F_1_ population (*n* = 182). Marker distribution of 1053 single‐nucleotide polymorphisms across 16 linkage groups (LG). (B) Scatter plot showing the relationship between the F_1_ genetic map position (cM) and physical genome position (Mbp) of 1053 single‐nucleotide polymorphisms for the 16 linkage groups. Linkage groups 1–16 correspond to the 16 physical chromosomes of the pecan genome, respectively.

A linkage map was constructed for the F_1_ population (*n* = 182), with 1053 SNPs anchored to the map (Table S6). The linkage map was successfully resolved into 16 LGs, corresponding to the haploid chromosome number of pecan, spanning a total genetic distance of 1639.2 cM (Figure 4A; Table S7). Individual LG lengths showed considerable variation, ranging from 62.3 to 143.4 cM, with an average of 102.5 cM per group. The average marker per cM across these LGs was 0.64, with the highest marker per cM of 0.85 on LG 7 and the lowest of 0.44 on LG 14. The max gap (21.6 cM) was observed at LG 6. When comparing the genetic map with the physical map, there are clear plateaus in most of the chromosomes where the cM does not increase but the Mbp does, indicating regions where recombination is repressed (Figure 4B). The overall distribution of markers across the 16 LGs was well‐balanced, suggesting comprehensive genome coverage and providing a robust framework for future genetic studies, QTL mapping, and MAS in pecan breeding programs.

## 4 DISCUSSION

### 4.1 Availability and access to the DArTag panel

The pecan DArTag panel is now openly available for any researcher or breeder to order through DArT (https://www.diversityarrays.com/). The panel was designed to produce amplicons of a minimum of 81 bp in length. Raw data in FAST‐All with quality score can be requested as can the MADC that indicates the read depth of each microhaplotype in each sample. The pecan 3K DArTag panel demonstrated high amplification rates, with a missing rate of less than 15% at sample level across the entire F_1_ population and the diversity accessions. The high detection rate and repeatability made this panel suitable and reliable for genetic map construction, MAS, association studies, genomic selection, reconstruction of recombination patterns, allele dosage estimation, and parental confirmation in North American cultivated pecan accessions, with some limited application in other *Carya* species that were not extensively tested in this study. The efficacy of the panel on breeding materials outside of North America also has not been tested at this time. The marker quality parameters of the DArTag panel for pecan are also comparable to those obtained for 3K DArTag panels for alfalfa (Zhao et al., 2023), blueberry (Zhao, Sapkota, et al., 2024), sweetpotato (Zhao, Sandercock, et al., 2024), and cranberry (Chen et al., 2025).

### 4.2 Utility of DArTag genotypic data in pecan breeding applications

Molecular marker technologies facilitate the genetic characterization of numerous genotypes through simple and cost‐effective procedures. Detecting polymorphisms is crucial for selecting molecular markers in genetic studies (Serrote et al., 2020). The PIC values for pecan have been evaluated using various marker systems, including RAPD markers (mean PIC = 0.740) in 30 pecan genotypes (Kaur et al., 2017), expressed sequence tag‐SSR markers (mean PIC = 0.703) in 44 diverse accessions (Lou et al., 2023), SSR markers (mean PIC = 0.547) in 80 diverse accessions (Zhang et al., 2020), and indel markers (mean PIC = 0.348) in 34 pecan collections (Mo et al., 2024). In this study, PIC values assessed for DArTag loci ranged from 0.0001 to 0.825, with an average of 0.334 in the diverse population. In comparison, DArTag‐derived SNPs exhibited PIC values ranging from 0.0001 to 0.375, with an average of 0.182. These findings highlight the varying levels of polymorphism captured by each marker system, offering valuable insights for genetic diversity, utility, and breeding efforts in pecan.

Genetic linkage maps are essential tools for identifying QTL and major genes that control agronomically important traits. These identified loci can be directly applied in marker‐assisted breeding programs. Using the pecan 3K DArTag panel, a linkage map was also successfully constructed, resulting in a total length of 1639.2 cM. The total map length was compatible with the first two single‐parent linkage maps (2227 and 1698 cM) for pecan based on RAPD and AFLP markers (Beedanagari et al., 2005), and two single‐parent informative GBS‐based linkage maps (1376.4 and 1463.1 cM) for pecan, as well as their consensus map (1557.8 cM) (Bentley et al., 2020).

### 4.3 Workflow and costs

The DArTag assay has a 3‐week turnaround time, starting from gDNA or tissue at a cost of approximately $15 per sample through to data extraction. The genotyping data report comprises allele dose calls and raw data with custom report formats available upon request. One benefit that DArTag has over fixed array platforms is the ability to update and improve the panel as required over time. The panel is a pool of 3100 oligos, one per locus, which is used to generate the sequencing libraries from the assayed material. Because the pool is created from individual oligo stocks, the removal of suboptimal loci or the addition of new loci can be easily done by creating a new pool. To determine which loci should be considered for removal, extensive genotyping (>7000 samples) is underway to identify those loci that consistently underperform or fail and flag them for removal. Independently, as QTL markers and/or markers specific to other germplasm are detected, they can be targeted for inclusion in the original pool in the next version(s) of the panel. DArT offers re‐pooling services once per year at low or no cost, but more frequent requests could result in labor surcharges being applied (Andrzej Kilian, personal communication, 2023). Researchers interested in initiating projects with DArT are encouraged to contact DArT directly for consultation.

### 4.4 Future considerations

Another benefit of the deep testing underway is the ability to detect and catalog all the microhaplotypes into a fixed allele database, which will improve combining datasets across genotyping projects (manuscript in preparation). If, after deep testing, it is clear that there are too few markers for GWAS for the given traits of interest, additional panels can be made to complement this panel. The other option is to add the required loci to the existing panel up to the technical limit of 7K, which is a more cost‐effective option for the routine genotyping service with scalability.

We chose to create a panel of 3K loci due to budgetary and technical reasons, but smaller complementary panels can be made at lower up‐front and downstream usage costs. The addition of a complementary 3K panel would nearly double the cost of genotyping per sample but would result in more granular genotyping data.

## AUTHOR CONTRIBUTIONS

**Shufen Chen**: Data curation; formal analysis; investigation; methodology; writing—original draft; writing—review and editing. **Meng Lin**: Conceptualization; data curation; investigation; methodology; supervision; writing—original draft; writing—review and editing. **Dongyan Zhao**: Data curation; methodology; resources; supervision; writing—review and editing. **Warren Chatwin**: Conceptualization; data curation; funding acquisition; investigation; methodology; project administration; resources; writing—review and editing. **Xinwang Wang**: Data curation; investigation; methodology; resources; writing—review and editing. **Angelyn Hilton**: Data curation; investigation; methodology; resources; writing—review and editing. **John T. Lovell**: Data curation; formal analysis; resources; supervision; validation; writing—review and editing. **Jennifer Randall**: Conceptualization; investigation; resources; supervision. **Kasia Heller‐Uszynska**: Methodology; resources; validation; writing—review and editing. **Cristiane H. Taniguti**: Software; validation; visualization; writing—review and editing. **Craig T. Beil**: Project administration; resources; supervision; writing—review and editing. **Moira J. Sheehan**: Conceptualization; funding acquisition; project administration; resources; supervision; writing—review and editing.

## CONFLICT OF INTEREST STATEMENT

The authors declare no conflicts of interest.

## Supporting information




**Supplemental Figure S1** A) Filters and criteria applied to create the pecan 3K DArTag marker panel. M, millions; K, thousands; B) Distribution of the 3100 DArTag markers across the pecan genome. The red bars represent the 3100 loci in physical position on the 16 chromosomes (grey bars).
**Supplemental Figure S2** Scatter plot showing the genetic relationship between offspring and each of the two parents in the F_1_ population. Six offspring inside the black polygon were excluded from the linkage map construction as suspicious potential outliers.
**Supplemental Figure S3** Scatter plot of polymorphism information content (PIC) values of 2968 DArTag loci using microhaplotypes and target SNPs in the diverse population.

**Supplemental Table S1**. The first three principal components using read counts from the DArTag Missing Allele Discovery Counts report and accession information for tested pecan samples.
**Supplemental Table S2**. Number of samples within various marker detection rates in the 376 pecan samples genotyped.
**Supplemental Table S3**. Sample‐based missing rates of the testing materials for the 3K pecan DArTag panel.
**Supplemental Table S4**. Polymorphism information content (PIC) values for DArTag loci using read counts of microhaplotypes in the diverse population.
**Supplemental Table S5**. Polymorphism information content (PIC) values of single nucleotide polymorphisms (SNPs) using read counts in the diverse population.
**Supplemental Table S6**. Linkage map constructed for the F_1_ population and genomic information of single nucleotide polymorphisms (SNPs) included in the linkage map.
**Supplemental Table S7**. Summary statistics of the linkage map constructed for the F_1_ population.

## Data Availability

The code and data are available in our GitHub repository for those interested in reproducing our analysis (https://github.com/Breeding‐Insight/Pecan_DArTag_Panel_paper).
