# Supplementary material for: A public mid‐density genotyping platform for pecan [Carya illinoinensis (Wangenh.) K. Koch]
Source: Plant Genome. 2026 Jun 13;19(2):e70262. doi: 10.1002/tpg2.70262 (PMC13263899; doi:10.1002/tpg2.70262)
Supplement: Supplementary file 1 [file TPG2-19-e70262-s001.docx]

**Supplemental Figure S1** A) Filters and criteria applied to create the pecan 3K DArTag marker panel. M, millions; K, thousands; B) Distribution of the 3100 DArTag loci across the pecan genome. The red bars represent the 3100 loci in physical position on the 16 chromosomes (grey bars).

A

B

**Supplemental Figure S2** Scatter plot showing the genetic relationship between offspring and each of the two parents in the F_1_ population. Six offspring inside the black polygon were excluded from the linkage map construction as suspicious potential outliers.

**Supplemental Figure S3** Scatter plot of polymorphism information content (PIC) values of 2,968 DArTag markers using microhaplotypes and target SNPs in the diverse population.
