# Supplementary material for: A public mid‐density genotyping platform for pecan [Carya illinoinensis (Wangenh.) K. Koch]
Source: Plant Genome. 2026 Jun 13;19(2):e70262. doi: 10.1002/tpg2.70262 (PMC13263899; doi:10.1002/tpg2.70262)

**Supplemental Figure S1**A) Filters and criteria applied to create the pecan 3K DArTag marker panel. M, millions; K, thousands; B) Distribution of the 3100 DArTag loci across the pecan genome. The red bars represent the 3100 loci in physical position on the 16 chromosomes (grey bars).

A


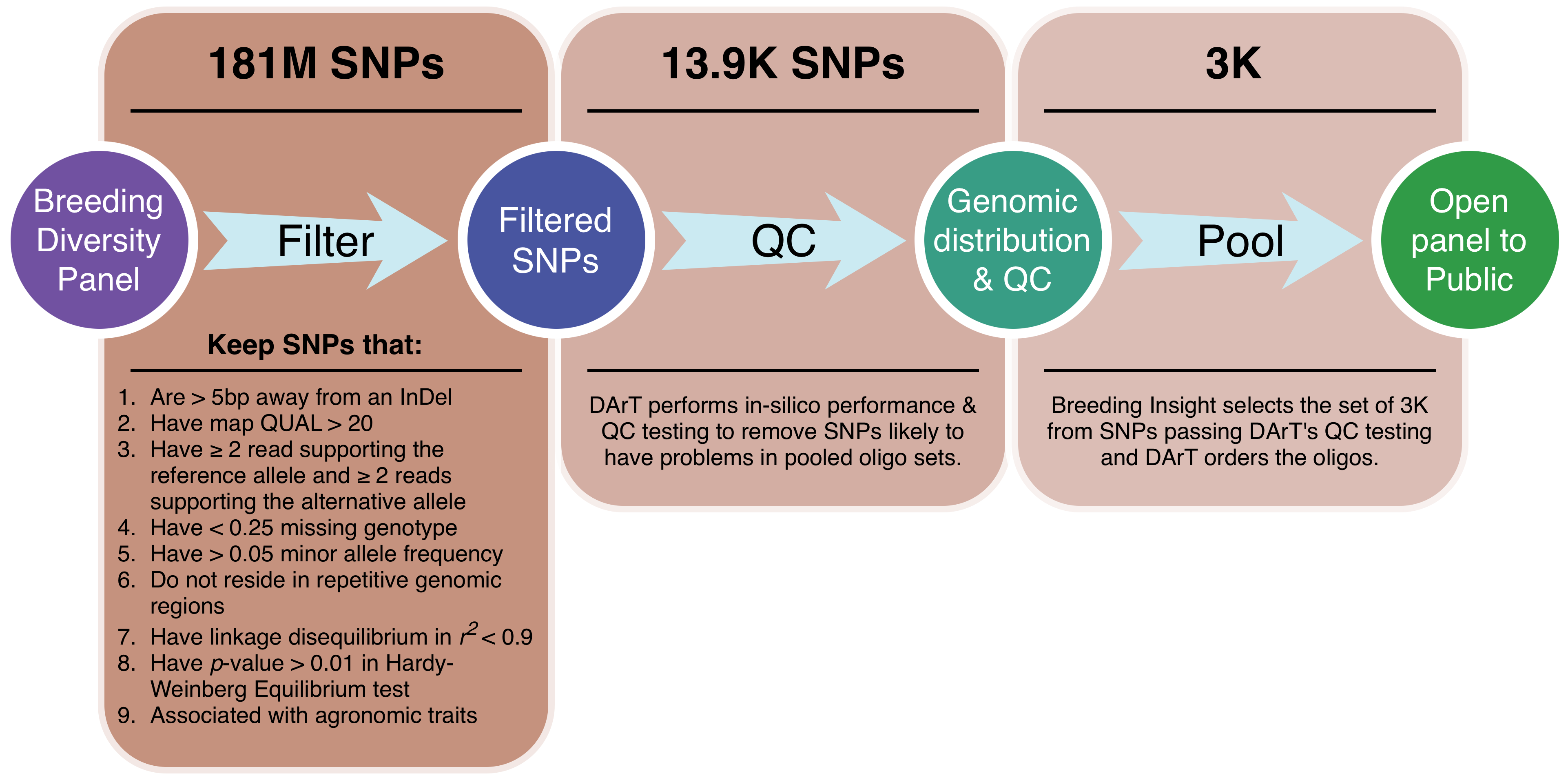


B

**
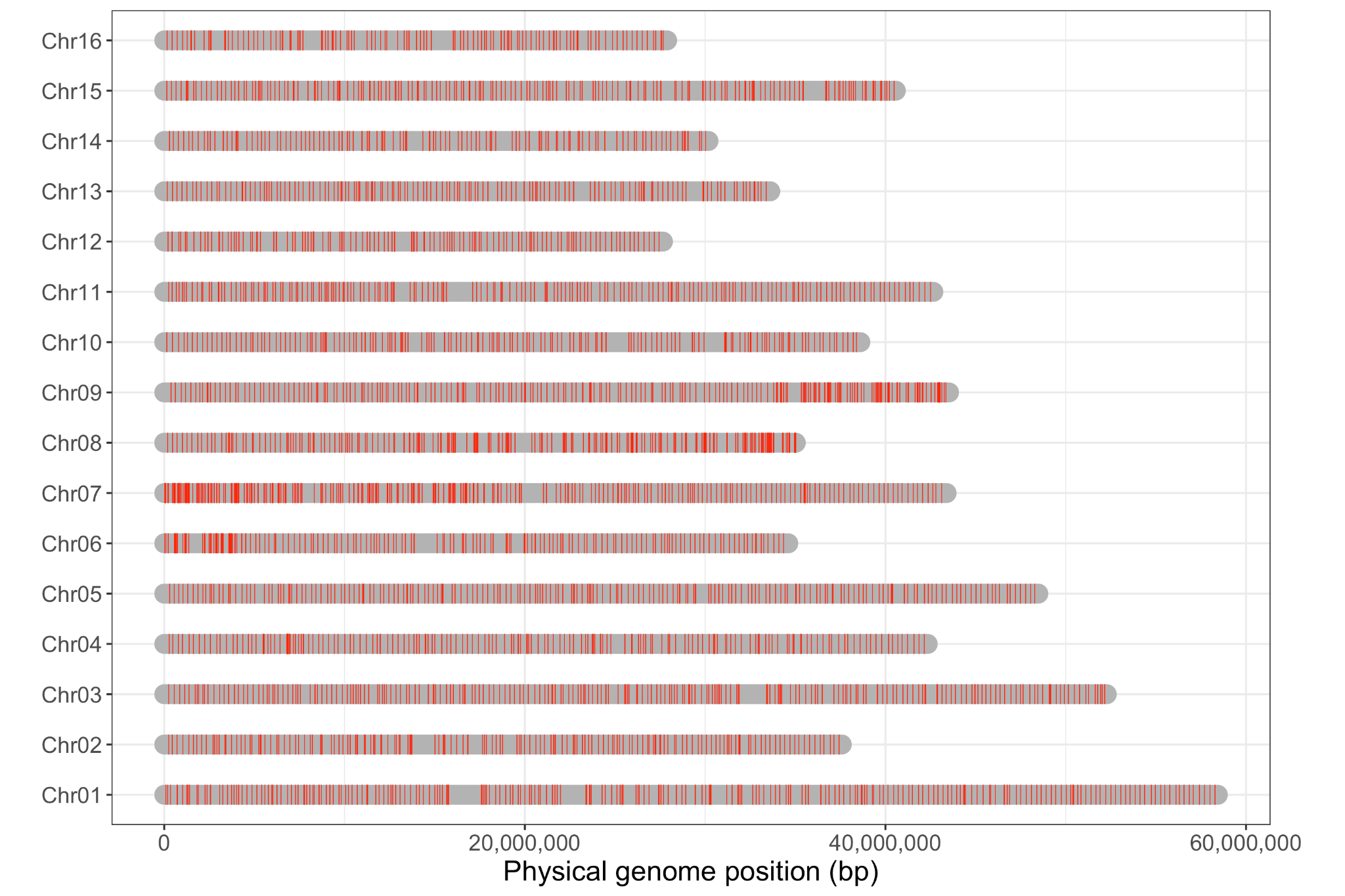
**

**Supplemental Figure S2**Scatter plot showing the genetic relationship between offspring and each of the two parents in the F_1_ population. Six offspring inside the black polygon were excluded from the linkage map construction as suspicious potential outliers.

**
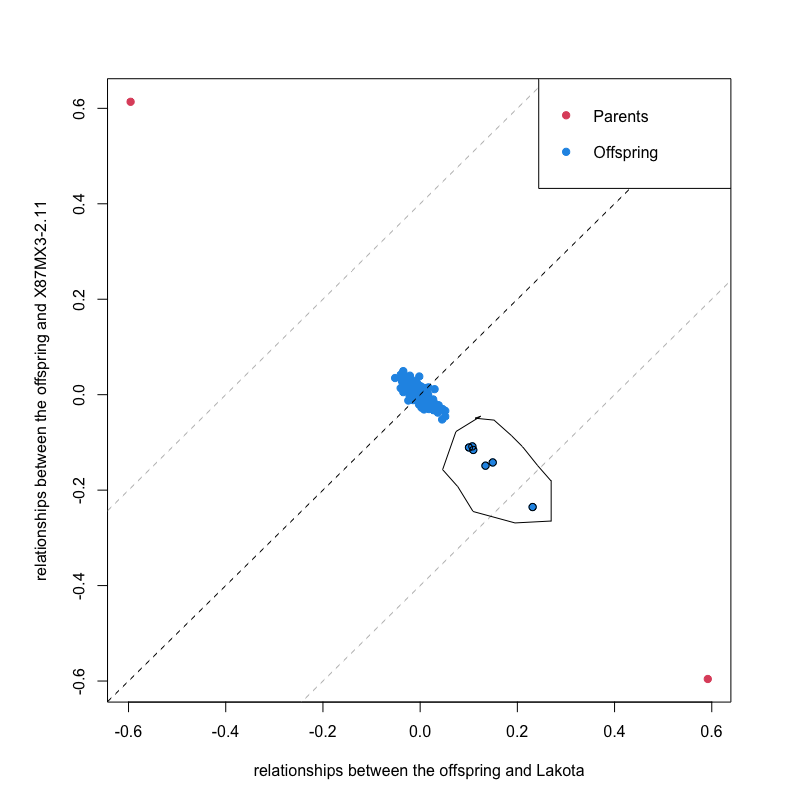
**

**Supplemental Figure S3** Scatter plot of polymorphism information content (PIC) values of 2,968 DArTag markers using microhaplotypes and target SNPs in the diverse population.


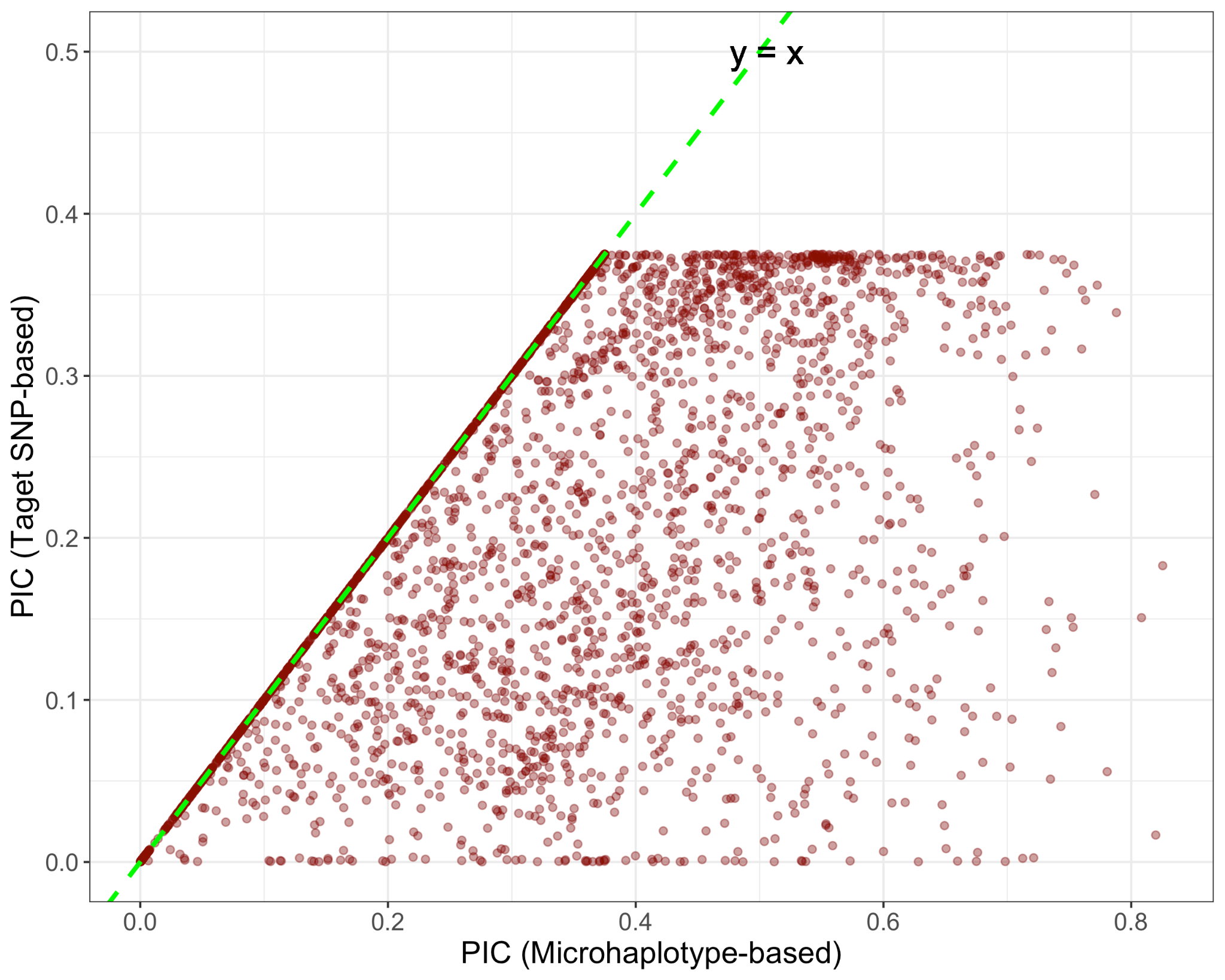

Supplement: Supplementary file 1 — Supplemental Figure S1 A) Filters and criteria applied to create the pecan 3K DArTag marker panel. M, millions; K, thousands; B) Distribution of the 3100 DArTag markers across the pecan genome. The red bars represent the 3100 loci in physical position on the 16 chromosomes (grey bars). Supplemental Figure S2 Scatter plot showing the genetic relationship between offspring and each of the two parents in the F1 population. Six offspring inside the black polygon were excluded from the linkage map construction as suspicious potential outliers. Supplemental Figure S3 Scatter plot of polymorphism information content (PIC) values of 2968 DArTag loci using microhaplotypes and target SNPs in the diverse population. [file TPG2-19-e70262-s001.docx]
